# Infectious Agents and Neurodegeneration

**DOI:** 10.1007/s12035-012-8320-7

**Published:** 2012-08-17

**Authors:** Giovanna De Chiara, Maria Elena Marcocci, Rossella Sgarbanti, Livia Civitelli, Cristian Ripoli, Roberto Piacentini, Enrico Garaci, Claudio Grassi, Anna Teresa Palamara

**Affiliations:** 1 Department of Cell Biology and Neuroscience, Istituto Superiore di Sanità, Rome, Italy; 2 Department of Public Health and Infectious Diseases, Sapienza University of Rome, Rome, Italy; 3 San Raffaele Pisana Scientific Institute for Research, Hospitalization and Health Care, Rome, Italy; 4 Institute of Human Physiology, Università Cattolica, Rome, Italy; 5 Department of Experimental Medicine and Biochemical Sciences, University of Rome “Tor Vergata”, Rome, Italy; 6 Department of Public Health and Infectious Diseases, Institute Pasteur Cenci Bolognetti Foundation, Sapienza University of Rome, Rome, Italy

**Keywords:** HSV-1, HIV, Influenza virus, *C. pneumoniae*, Alzheimer’s disease, Neurodegeneration

## Abstract

A growing body of epidemiologic and experimental data point to chronic bacterial and viral infections as possible risk factors for neurodegenerative diseases, including Alzheimer’s disease, Parkinson’s disease and amyotrophic lateral sclerosis. Infections of the central nervous system, especially those characterized by a chronic progressive course, may produce multiple damage in infected and neighbouring cells. The activation of inflammatory processes and host immune responses cause chronic damage resulting in alterations of neuronal function and viability, but different pathogens can also directly trigger neurotoxic pathways. Indeed, viral and microbial agents have been reported to produce molecular hallmarks of neurodegeneration, such as the production and deposit of misfolded protein aggregates, oxidative stress, deficient autophagic processes, synaptopathies and neuronal death. These effects may act in synergy with other recognized risk factors, such as aging, concomitant metabolic diseases and the host’s specific genetic signature. This review will focus on the contribution given to neurodegeneration by herpes simplex type-1, human immunodeficiency and influenza viruses, and by *Chlamydia pneumoniae*.

## Introduction

Neurodegenerative diseases, including Alzheimer's disease (AD), Parkinson's disease (PD), Huntington's disease (HD) and amyotrophic lateral sclerosis (ALS), are devastating pathologies characterized by progressive degeneration and loss of specific subsets of neurons that lead to a decline in brain functions such as cognition and locomotor control. Although these diseases have very different clinical manifestations, depending partly on which region of the brain is affected, they share some common features and pathological hallmarks, including the formation and deposition of aberrant protein conformers, synaptic dysfunctions, oxidative stress, deficient autophagic processes, and inflammation.

The causative agents of these highly complex diseases, which are often the result of several combined genetic and environmental factors, are still unknown and the molecular basis underlying their pathogenesis has yet to be fully clarified. Starting from the pioneer study by Bowery [1] showing the neurodegenerative effects produced by tetanus toxin in rats, a significant emerging body of literature suggests the possibility that CNS infections may play a cofactorial role in inducing neurodegenerative diseases [2]. Serological studies suggest a potential involvement of enteroviruses (EV) and human herpesviruses (HHV) in the aetiology of ALS, a fatal neurodegenerative disease selectively affecting motor neurons [3]. A viral origin of PD has also been proposed on the basis of the close similarity between the clinical symptoms of PD and those of Japanese encephalitic virus [4]. Ever since the earliest papers describing the neurological symptoms associated with influenza [5, 6], the influenza virus has repeatedly been suggested as an aetiological agent for PD [7]. Several findings also support the involvement of infectious agents (in particular herpes simplex virus type-1 [HSV-1] and *Chlamydia pneumoniae*) in the pathogenesis of AD, a multifactorial disorder characterized by severe memory impairment and cognitive decline that affects hippocampal and basal cortex neurons [8–15]. Among the human herpesviruses, herpes simplex virus, Epstein Barr Virus, varicella zoster virus, cytomegalovirus, human herpesvirus-6 and, more recently, herpesvirus-7 have all been associated with multiple sclerosis (MS), an inflammatory disease leading to demyelization of nerve cell axons in the spinal cord and brain [16–23].

Some of these correlations are not yet supported by conclusive experimental and clinical evidence, but a growing body of data supports the hypothesis that chronic damage induced by different infectious agents may concur to produce neurodegeneration. Obviously, the long-term effects of persistent or lifelong repeated infections may differ in different hosts, according to their general health, pharmacological treatments, genetic background, concurrent diseases, etc. Even considering the different outcomes due to these conditions, several findings indicate that pathogen-related long-term damage may underlie several neuronal dysfunctions typical of aging [2].

In this regard, it is worth noting that the CNS may be particularly vulnerable to infectious agents during aging on account of alterations to the blood–brain barrier (BBB), as well as age-related increased oxidative stress and impaired energy production [2]. Aged neurons are certainly more vulnerable to many insults, including the toxicity of viral or prion proteins, due to increased oxidative stress and impaired neurotrophic factor signalling pathways [24, 25].

Although activation of the host immune response in an attempt to eradicate the pathogen [26] may significantly contribute to produce neuronal damage, different pathogens and/or their products may directly induce long-term degenerative effects, such as the deposit of misfolded protein aggregates, increased levels of oxidative stress, deficient autophagic processes, synaptopathies and neuronal death. This review will focus on the degenerative effects of chronic or persistent infections caused by herpes simplex virus, human immunodeficiency virus, influenza virus and *C. pneumoniae*.

## Route of Entry of Infectious Agents into the CNS

**Fig. 1:**
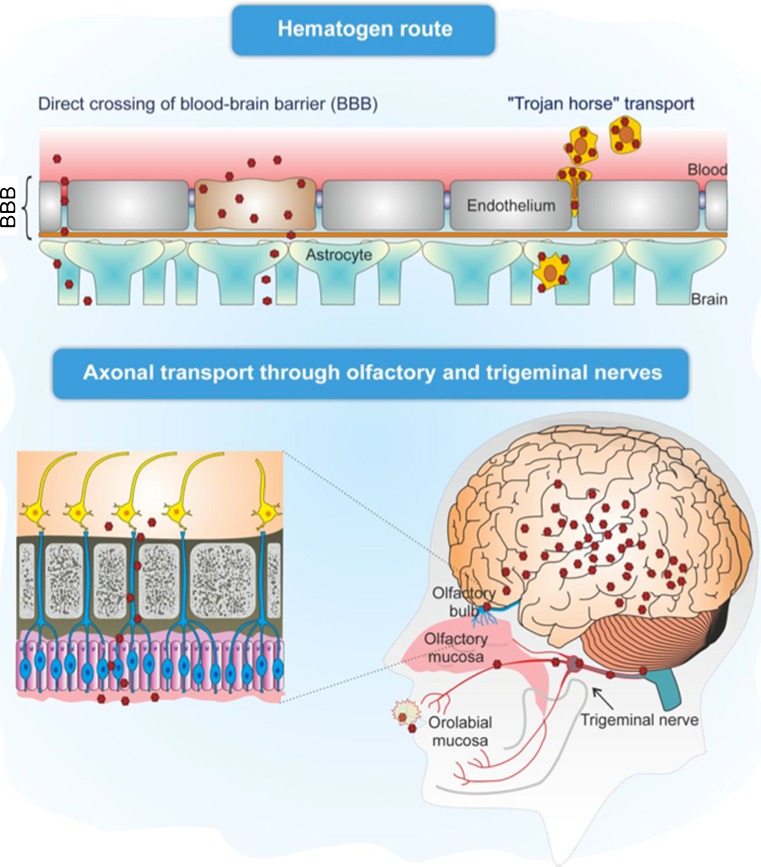
Infectious agents can reach the central nervous system by either crossing the blood–brain barrier (hematogen route) or being transported by axons of cranial nerve neurons (for further details, see text)

Viruses, bacteria, protozoa and unconventional pathogens such as prion proteins have the ability to invade the CNS and cause acute infections which in some cases may be fatal or which may progress to become chronic illnesses [26, 27]. Here, we focus on selected human viruses and bacteria to describe various mechanisms of neuroinvasion (Fig. 1).

In immunocompetent subjects with a fully functional BBB very few pathogens are able to infect the brain through the blood. Indeed, the BBB and cerebral spinal fluid (CSF) barrier prevent the unselective diffusion of vascular and cellular components. The BBB, in particular, is composed mainly of non-fenestrated endothelial cells interconnected by tight junctions in contact with astrocyte processes. Besides limiting the movement of cells and molecules to the brain parenchyma and neurons, these cells also provide a physical and cellular barrier to the perivascular space by producing a basement membrane consisting of laminin. However, some pathogens are able to disrupt the BBB and to cross directly into the CSF through the porous capillaries of the choroid plexus, thus passing into the brain and provoking severe haemorrhagic encephalitis. When the BBB is compromised, as in many diseases, different viruses can enter the brain through the bloodstream.

Other possible routes of entry into the CNS are: (1) infection of cells belonging to the monocyte–macrophage/microglia lineage that are able to cross the BBB; (2) intra- and trans-neuronal transfer from peripheral neurons.

1. Viruses of the lentivirus family, which includes HIV, cross the BBB through the “Trojan Horse” mechanism [28, 29], driven by infected leukocytes, which allow the virus to escape the immune system and to move from the bloodstream to the brain. Inflammation enhances this invasion of the CNS, as inflammatory molecules released during the systemic infection activate infected leukocytes, which in turn attach to and invade the postcapillary venule wall surrounding endothelial and parenchymal basement membranes, thus crossing the BBB. Leukocytes are further activated by interactions between chemokine receptors expressed on their membranes and chemokines circulating in the brain. For example, increased expression of chemokine ligand 2 (CCL2) by endothelial cells and astrocytes following contact with HIV-infected cells [30], together with virus-induced alterations of endothelial adhesion molecules and junctional proteins [31], amplifies disruption of the BBB and viral entry. Moreover, dysregulation of BBB, likely caused by a combination of viral and host factors (e.g., secreted viral proteins, inflammatory mediators, antiviral therapies, drug or alcohol abuse, aging), has been proposed as a critical component of HIV-induced neuropathology [32]. Virus-bearing monocytes then infect perivascular macrophages and microglia, but not neurons, which are, however, negatively affected by the release of inflammatory cytokines and/or viral proteins by neighbouring cells.
2. The peripheral nerve endings located in the skin and the mucosa mediate the other mechanism of entry of viruses that infect the CNS. Depending on the differential expression of viral receptors, a particular neurotropic virus will target a specific type of peripheral nerve ending, including those of sensory, motor and olfactory neurons. Viruses infecting these sites exploit cellular components such as the axonal microtubules and microtubule-based motor proteins [33] to move, through retrograde axonal transport, from the cell periphery to the neuronal cell body that contains the synthetic machinery exploited for viral replication, as well as to return through anterograde transport to the cell periphery and spread into target cells [34].

HSV-1, for example, can infect oral and nasal mucosa and then travels through retrograde axonal transport to the trigeminal ganglion or the olfactory bulb, respectively, where it establishes a latent infection or may rapidly enter the CNS [35]. It is worth noting that HSV-1 infection has been reported to suppress the induction of neuronal apoptosis in the olfactory neuroepithelium and trigeminal and dorsal root ganglia in order to facilitate neuroinvasion of the brain [36, 37]. Periodic reactivations from latency are followed by axonal transport of newly produced HSV-1 virions either back to the site of primary infection, where they cause new skin vesicles or mucosal ulcers, or onward to the CNS, where they can cause a productive, but usually mild infection, which may later become latent, as described for rodents [38, 39]. In particular, newly produced virions may target the limbic system, which includes the hippocampus, thalamus and amygdala [40]. Indeed, in HSV encephalitis, the major site of damage is the limbic system [41] that is presumably reached through the olfactory bulb, since HSV-1 can infect cells in the nasal endothelium [42, 43].

The olfactory system provides a bridge between the peripheral environment and the brain and several other neurotropic viruses, including rabies, influenza A virus and parainfluenza virus are known to infect the CNS by transmission through the olfactory pathway. The A/WSN/33 strain of influenza virus has been shown to enter the CNS via the olfactory epithelium [44]. However, it has also been hypothesized that it can enter the CNS via other cranial nerves, including the vagus, and trigeminal nerves [45–48]. These nerves have processes that innervate visceral organs and tissues that are thought to be initially targeted by intranasal viral infection, including the olfactory epithelium (olfactory nerve, CN I), orofacial mucosa (trigeminal nerve, CN V) and digestive system (vagus nerve, CN X). Accordingly, the virus has been found in the regions innervated by these nerves following infection of animal models via intranasal routes. The virus has also been detected (by immunohistochemical staining of viral nuclear protein, NP) in the visceral ganglia [48, 49]. However, evidence that the A/WSN/33 strain of influenza has an affinity for the substantia nigra [50], a neuronal population with no direct anatomical connection to the cranial nerve system, suggests that other routes of invasion may be involved. In particular, it is possible to hypothesize that it can move into the brain through the ependymal cells lining the ventricles and shedding into the CSF, where it can freely spread throughout the neuraxis; through the extravasation from capillaries that penetrate into the brain; or through direct invasion of the CNS after BBB disruptions.

After entering the CNS, viruses promote cell-to-cell dissemination through different mechanisms, i.e. release into the synaptic cleft or via fusion events with neighbouring neurons. They may also reinfect peripheral tissue. Alphaviruses use the anterograde transport system to move from the cell body to the axonal terminal where they are released by exocytosis into the synaptic cleft [35]. However, during anterograde transport, the virus can also exit through axonal varicosities before reaching the termini and infect neighbouring cells [51, 52].

Invasion of the CNS by pathogens may result in acute infection, possibly followed by latency and reactivations, or in persistent infection causing chronic damage that accumulates with time. The occurrence of each outcome may depend on several factors, including the type of pathogen, the kind of host immune/inflammatory response elicited, the CNS region affected, the general conditions of the host and the presence of concomitant diseases.

## Epidemiological Evidence

### HSV-1 and Alzheimer's Disease

**Fig. 2:**
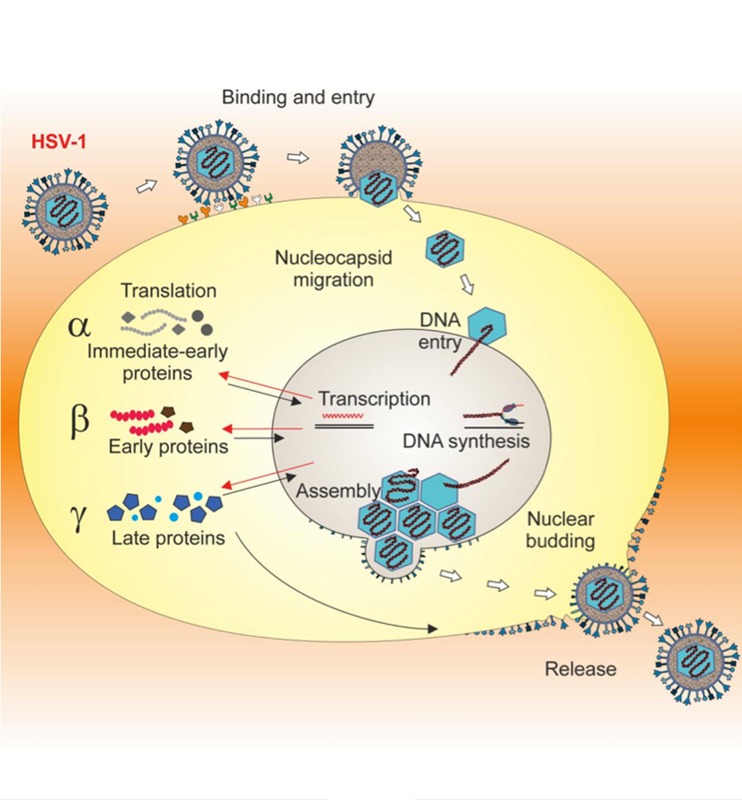
HSV is a double-stranded DNA virus, structurally composed of a linear genome packaged into an icosahedral capsid enclosed by tegument proteins and surrounded by a lipid bilayer membrane with embedded proteins and glycoproteins (envelope). Primary infection in humans usually occurs in the orofacial mucosa during childhood. There the virus replicates within the epithelial cells and undergoes its typical lytic life cycle ending with the production of infectious virions and lysis of the host cell. HSV entry into the host cell requires sequential interaction between specific viral membrane glycoproteins (gB, gC, gD, gH and gL) and cellular receptors [heparan sulphate proteoglycans (HSPG), nectin-1 and 2, herpesvirus entry mediator (HVEM) or 3-O sulphated heparan sulphate (3-OS HS)]. On entry, the nucleocapsid is transported to the nuclear membrane and the viral DNA is released into the nucleus for transcription of viral genes and replication. The HSV genome consists of two long structures of unique sequences (designated long (UL) and short (US)), that encode over 80 distinct genes, and it is transcribed by the RNA polymerase II of the infected host. Immediate-early genes are the first to be expressed following infection and they encode proteins that regulate the subsequent expression of *early* and *late* viral genes. Early gene expression then allows the synthesis of enzymes involved in DNA replication and the production of certain envelope glycoproteins. Expression of late genes occurs last; this group of genes predominantly encodes proteins that form the virus particle. This latter is then released from host cells by budding. HSV-1 is able to establish a lifelong latent infection in sensory neurons, particularly in cellular bodies of those feeding the site of primary infection [53]. This latency is characterized by the presence of a functional viral genome without production of the infectious virus. During this time, the latency-associated transcripts (LATs) are the only prominent transcripts [54] whose role in generating functional peptides or proteins is still a matter of debate [55]. Reactivation from latency can be triggered by several external stimuli (stress, immunosuppression, etc.) that activate viral gene expression. Newly produced virions are transported to the sites of primary infection where they cause recurrent herpetic lesions in some people. Interestingly, APOE4 is a risk factor for these recurrent herpetic lesions [56, 57]

Chronic and persistent exposure to HSV-1 (see Fig. 2) has been proposed as a potential risk factor for AD. HSV-1 is a ubiquitous neurotropic virus that affects between 56 and 85 % of the world population, with country-to-country variations, and more than one third of the population has recurrent clinical HSV-1 infections and manifestations. Interestingly, epidemiological studies have reported the presence of the HSV-1 genome in post-mortem brain specimens from numerous AD patients, particularly those who carry the type 4 allele of the gene that encodes apolipoprotein E (APOE4), another potential risk factor for AD [14, 56]. Moreover, genes related to HSV-1 reactivation have been detected in the brain of patients with familial AD, associated with β-amyloid deposits [13], and HSV-1 DNA has been found in amyloid plaques from the temporal and frontal cortices of AD sufferers [58]. Recently, a large prospective population-based study also showed that the risk of AD is increased in elderly subjects with positive titers of anti-HSV-1 IgM antibodies, which are markers of primary or reactivated HSV-1 infections, while it is not associated with anti-HSV-1 IgG antibodies, which are markers of a life-long infection [59]. Finally, genome-wide association (GWA) studies have correlated individual brain susceptibility to HSV-1 infection with a genetic risk of AD [60, 61]. In particular, analysis of data from GWA studies of several thousand European AD patients and controls [60] identified a set of AD-linked gene variants that may increase the brain's susceptibility to viral infections [61]. These include: nectin-2, also known as herpes virus entry-mediator-B or poliovirus receptor-related protein-2, which mediates the entry of HSV into host cells; apolipoprotein E (APOE), particularly its ε4 allele, which besides being a well-estabilished genetic risk factor for AD, has also been shown to influence susceptibility to viral infections and spreading into neuronal cells; translocase of the outer mitochondrial membrane 40 homolog (TOMM40), whose variations might influence mitochondrial damage induced by HSV DNAase such as UL12.5, and other genes.

These variants form a genetic signature that may determine individual brain susceptibility to HSV-1 infection during aging or susceptibility to pathogen-driven damages, particularly those leading to neurodegeneration.

### *C. pneumoniae* and AD

**Fig. 3:**
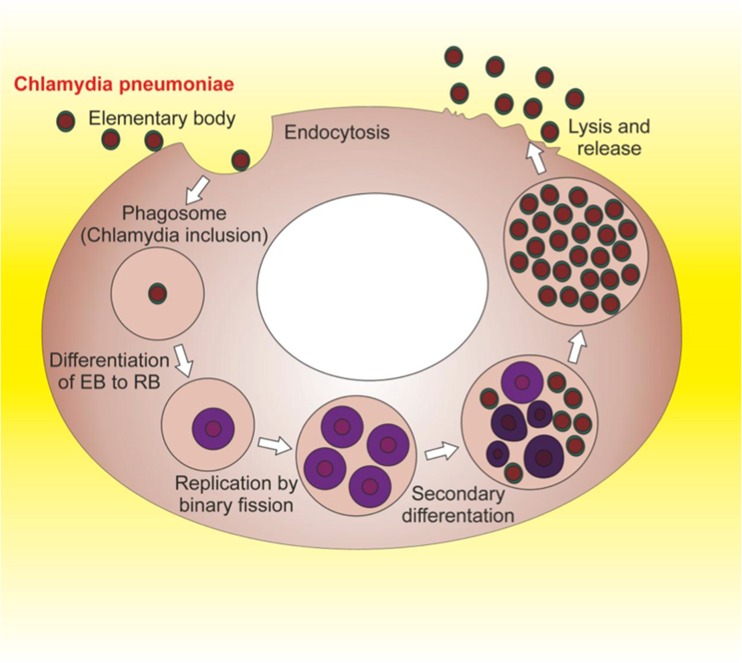
*Chlamydiae* are Gram-negative bacteria. They are obligate intracellular parasites because their multiplication depends on the host cell for energy and various nutrients. *Chlamidiae* have evolved a unique biphasic developmental cycle in which they alternate two distinct morphological forms: the elementary body (*EB*) and the reticulate body (*RB*). EBs are small tight bodies and represent the metabolically inactive form of bacteria, which can resist environmental stress and survive outside a host for a limited time. Infection begins with the attachment of the EB to the surface of susceptible host cells, followed by its internalization by endocytosis and the formation of phagosomes (*Chlamydi*a inclusions) that are heavily modified by chlamydial proteins which prevent their fusion with lysosomes. Shortly after uptake, EBs differentiate into the metabolically active form of RB and begin to replicate within the phagosomes. RBs replicate by binary fission that, after 24–72 h, becomes asynchronous, with some RBs converting back to EBs. Finally, EBs are released from infected cells, often after causing the death of the host cells, and can infect new cells, either in the same organism or in a new host. *C. pneunoniae* was classified as the third species of *Chlamidia* and was associated in humans with acute infections of the lower respiratory tract. It is recognized as a common cause of mild pneumonia in children and young adults. It infects both epithelial cells and macrophages within the lungs and may be disseminated to sites outside of the lungs by infected monocytes and macrophages. Infection may also persist or, alternatively, the bacterium may be present in asyntomatic patients. Recently, a large volume of research showed evidence that *C. pneumoniae* may contribute directly and indirectly (immuno-mediated) to atherosclerosis. Indeed, it was one of the few infectious agents that have been found within and isolated from cells of human atherosclerotic plaques

*C. pneumoniae* (see Fig. 3) infection was first linked to AD on the basis of evidence that a high percentage (90 %) of AD brains were found to be PCR-positive for this pathogen, particularly in the cerebral regions most affected by AD [8]. In particular, this microorganism, which is able to infect microglia, astrocytes, perivascular macrophages and monocytes [8, 62], was isolated from the tissue as metabolically active and propagated in cells. Other studies failed to detect *C. pneumoniae* in archival tissue of AD patients [63–65], but it has to be underlined that two of these studies were performed on tissue that was paraffin embedded, which may have affected the identification of the organism using the specific PCR technique [63, 65]. Other authors have demonstrated the presence of *C. pneumoniae* in AD patient brains through PCR analysis of frozen tissue [66]. More recently, Little et al. demonstrated that intranasal inoculation of *C. pneumoniae* in mice induced AD-like hallmarks in brains [67]. Moreover, *C. pneumoniae* antibodies have been identified in AD brains, colocalizing with plaques and tangles in vulnerable brain regions [68].

### HIV and Dementia

**Fig. 4:**
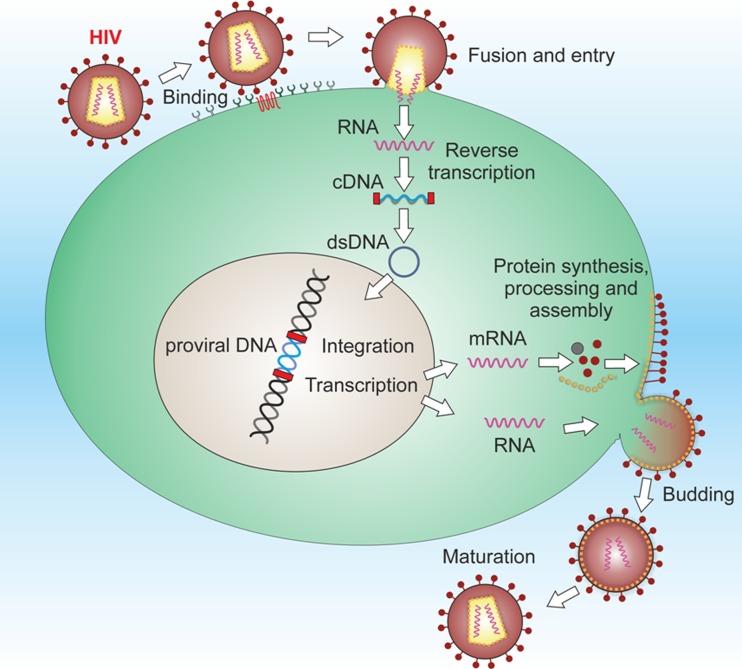
HIV-1 is an enveloped icosahedral retrovirus, belonging to the Lentivirus subgroup of *Retroviridae* family. Its genome is constituted by two identical copies of non-complementary positive single-stranded RNA, enclosed by a capsid composed of several copies of the viral protein p24. The single-stranded RNA is tightly bound to nucleocapsid proteins and enzymes needed for viral replication and assembly such as reverse transcriptase, proteases, ribonuclease and integrase. A matrix composed of the viral protein p17 surrounds the nucleocapsid and this is, in turn, surrounded by the viral envelope containing the surface glycoproteins gp120 and gp41 as protruding spikes. The HIV replication cycle begins with adsorption of the viral particles to CD4 molecules (a member of the immunoglobulin superfamily) on the surface of susceptible cells. The subsequent interaction with a co-receptor belonging to the family of chemokine receptors (CXCR4 or CCR5) plays a major role in membrane fusion and entry. Shortly after entry, subviral particles are partially uncoated in the cytoplasm and initiate the reverse transcription of viral RNA. The newly produced DNA is then transported into the nucleus and integrated into the host DNA by the virus-encoded integrase. The integrated HIV DNA is called provirus. The provirus may remain inactive for a long time, producing few or no new viral particles. The coordinated interaction of the HIV-encoded Tat protein and cellular transcription factors with the RNA polymerase II transcription apparatus starts the production of viral genomic RNA and messenger RNA, which is then spliced into smaller pieces, exported from the nucleus into the cytoplasm and translated into the proteins. In addition to viral structural proteins and enzymes, the HIV genome encodes the regulatory proteins (Rev and Tat, which is secreted by HIV-infected cells) and several accesory proteins: Vif, Vpu, Vpr, Vpx and Nef, playing an important role in the viral replication, disease pathogenesis and immune evasion. At the end of viral replication cycle, envelope polyproteins are transported to the plasma membrane where viral progeny begins assembly and budding from the infected cells. Then, subsequent proteolysis by viral protease generates mature particles

Almost 60 million people worldwide have been infected by HIV (see Fig. 4), a virus known for its devastating effects on the immune system, that results in acquired immune deficiency syndrome (AIDS), characterized by increased risk of several opportunistic infections and diseases. Although effective treatments for AIDS have prolonged the survival of infected patients, they have also resulted in a growing number of patients with neurological consequences of HIV infection [69, 70]. The virus can also cause severe neurological disorders, known as HIV-associated neurocognitive disorders (HAND), that are characterized by cognitive, motor and behavioral abnormalities and comprise: asymptomatic neurocognitive impairment (ANI), HIV-associated mild neurocognitive disorder (MND) and HIV-associated dementia (HAD). These pathologies are the result of the invasion of HIV and its replication within the CNS and consequent virus-induced degeneration of synapses and neurons in different brain regions, as well as associated neuroinflammation and immune activation of macrophages, microglia and astrocytes. Interestingly, APOEε4 has been proposed as a genetic factor for the development of a severe form of HAD, as it is for AD [71, 72]. Although the use of antiretroviral drugs, initially as monotherapy (single agents such as zidovudine) and then as combination therapy (highly active antiretroviral therapy or HAART), has changed the clinical management of HIV- and HAND-positive patients by suppressing the systemic viral load and consequently decreasing the mortality rates, the incidences of both opportunistic infections in AIDS patients and the most severe form of HAD, the prevalence of neurocognitive impairment remains high [70, 73–75]. One possible explanation is that HAART does not readily cross the BBB, making the CNS a safe haven for infection and permitting ongoing degenerative changes even when viral titers are low in the periphery. However, other factors have been suggested to be involved in modulating HAND pathologies, including the effects of aging on the vulnerability of the brain, the persistence of HIV replication in brain macrophages, the evolution of highly neurovirulent HIV strains affecting the CNS, and even long-term CNS toxicity due to HAART [reviewed in 76].

### Influenza Virus and Parkinson's Disease

**Fig. 5:**
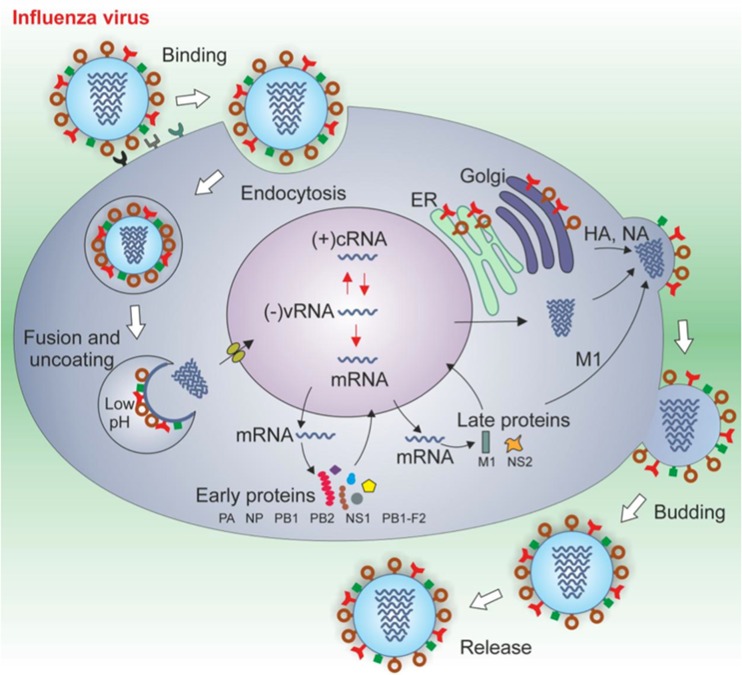
Influenza A viruses are enveloped, negative strand RNA viruses belonging to the *Orthomyxoviridae* family. Their genome consists of eight single-stranded RNA segments encoding 11 or 12 proteins: the receptor-binding haemagglutinin (*HA*); the sialic acid-destroying enzyme neuraminidase (*NA*), the ion channel M2, the matrix protein M1; the nucleoprotein (*NP*); the polymerase acidic protein (*PA*), polymerase basic proteins 1 and 2 (*PB1*, *PB2*) and the pro-apoptotic protein polymerase basic 1 (*PB1*)-F2; the nuclear export protein (*NEP*; also known as *NS2*) and the host antiviral response antagonist non-structural protein 1 (*NS1*); the newly identified N40 protein, which is expressed from the PB1 segment and has an unknown function [77]. Within the virion, each of the eight RNA segments forms a viral ribonucleoprotein (*RNP*) complex: in particular, viral RNA is wrapped around NP, and this structure is in turn bound to the viral polymerase complex, to constitute the viral nucleocapsid. In the initial stages of influenza A virus replication, the viral HA binds to host cell receptors that contain terminal α-2,6-linked or α-2,3-linked sialic acid (α-2,6-SA or α-2,3-SA) moieties, and the virus enters the cell by receptor-mediated endocytosis. Cleavage of HA by cellular proteases is required to expose the HA peptide that is responsible for the fusion between the viral envelope and the endosomal membrane. Acidification of the late endocytic vesicles allows the viral HA to undergo a conformational rearrangement that produces a fusogenic protein. The H^+^ ions in the acidic endosome are pumped, via the viral M2 ion channel, into the virus structure allowing the virus uncoating and the release of RNP complexes into the cytoplasm. The viral RNA is then imported in an ATP-dependent manner into the cell nucleus for transcription of genomic and messenger RNAs which are transported to the cytosol for translation. Viral HA, NA and M2 are synthesized in the Endoplasmic Reticulum, transported by the trans-Golgi secretory pathway and the mature proteins are inserted in the plasma membrane. New viral RNA is encased in the nucleocapsidic proteins and, together with matrix protein, is transported to cell surface where HA and NA will be incorporated. Progeny virions are then released from cells by budding

The influenza virus (see Fig. 5) has been implicated as both a direct and an indirect cause of PD, on the basis of both clinical descriptions and epidemiological studies. However, the link with PD is somewhat controversial. Much of the association of Parkinsonism with influenza and many other viruses stems from an outbreak of encephalitis lethargica (EL) (von Economo's disease) and the postencephalitic Parkinsonism that occurred subsequent to the 1918 influenza pandemic caused by a type A H1N1 influenza virus [78, 79, reviewed in 80]. Although the hypothesis that EL was a complication of influenza is supported by several data, it is still a matter of debate. One piece of evidence against the role of influenza as an agent of PD is the absence of viral RNA recovered from the brains of postencephalitic PD patients [81, 82], the absence of any known mutations that would make the 1918 influenza virus neurotropic, and questions regarding the timing of the 1918 pandemic waves and the outbreak of EL. Despite the apparent strength of the direct evidence against the influenza hypothesis, there is a strong epidemiological tie, based mostly on the increased incidence of PD in the wake of the 1918 H1N1 influenza A pandemic [83–85]. It has even been shown that persons born during the 1918 influenza pandemic had a 2–3-fold higher risk of Parkinson's disease than those born prior to 1888 or after 1924 [86, 87]. Poskanzer and Schwab [84] also showed an increase in PD onset based on an external event occurring around 1920. Recently, a large observational study carried out by Toovey et al. [88] using a large database from the UK, identified 3,976 cases of PD and 18,336 of Parkinson symptoms (PS) between 1994 and 2007 and concluded that influenza infections are associated with transient neurological sequelae such as tremor or gait disturbances. Interestingly, the risk of developing PS increases with the number of influenza attacks, suggesting that influenza-associated neuronal injury may be a cumulative process. The relative risk of developing neurological PS was highest within the first few weeks after a diagnosed and recorded influenza infection.

## Infectious Agents and Neurodegenerative Processes

### Protein Misfolding in the Brain

Numerous neurodegenerative disorders share common features, including protein aggregation and the formation of inclusion bodies or aggregate deposits in selected brain regions. These deposits usually consist of insoluble fibrillar aggregates containing misfolded protein with β-sheet conformation. Although the distribution and composition of these protein aggregates are different in each neurodegenerative disease, they show similar morphological, structural and staining characteristics [89] and represent typical disease hallmarks. Two types of protein deposit characterize AD: (1) amyloid plaques, comprising mainly 40- to 42-residue peptides named β-amyloid peptides (Aβs), are deposited extracellularly in the brain parenchyma and around the cerebral vessel wall [90]; (2) neurofribillary tangles containing paired helical filaments, composed mainly of hyperphosphorylated tau protein, accumulate in the cytoplasm of degenerating neurons [91]. The aggregates that characterize PD consist mainly of a protein named α-synuclein (α-SYN), which accumulates in Lewy bodies in the cytoplasm of substantia nigra neurons [92]. In HD patients, intranuclear deposits of a polyglutamine-rich version of huntingtin protein are found in the striatum [93]. SLA is characterized by aggregates composed mainly of superoxide dismutase in cell bodies and axons of motor neurons [94]. Finally, brains with various forms of transmissible spongiform encephalopathies (TSE) are characterized by an accumulation of preotease-resistant aggregates of prion protein (PrP) [95].

Historically, lesions that contain aggregates were considered to be pathogenic. In effect, abnormal aggregates are found in the brain region most affected by each disease. Additionally, mutations in the genes coding for misfolded proteins cause familial forms of these diseases, characterized by an earlier onset and a more severe phenotype than the sporadic forms. Recently, several findings have suggested that the small intermediates, i.e. soluble oligomers, within the complex multistep process by which misfolded proteins assemble into inclusion bodies are the most toxic forms of these aberrant proteins. They appear to be able to affect normal cell activities, whereas aggregates may represent a cellular attempt to wall off potentially toxic material [96] or might function as a reservoir of the bioactive oligomers.

Amyloid peptides (Aβs), found in extraneuronal plaques in AD patients as well as in intraneuronal compartments, result from the amyloidogenic proteolytic cleavage of Amyloid Precursor Protein (APP) by the sequential action of β- and γ-secretase enzymes, and a significant body of evidence suggests that Aβ accumulations in AD are the result of an imbalance between Aβ production and Aβ clearance [97–99]. Mutations in genes encoding APP and presenilin, a component of the γ-secretase complex, have been associated with the familial form of AD, whose pathogenesis is undoubtedly linked to their involvement in Aβ overproduction. However, the causes of the latter in sporadic forms of the disease have yet to be fully clarified. Interestingly, some pathogens have been associated with or involved in Aβ accumulation.

#### HSV-1 and Protein's Aggregates

**Fig. 6:**
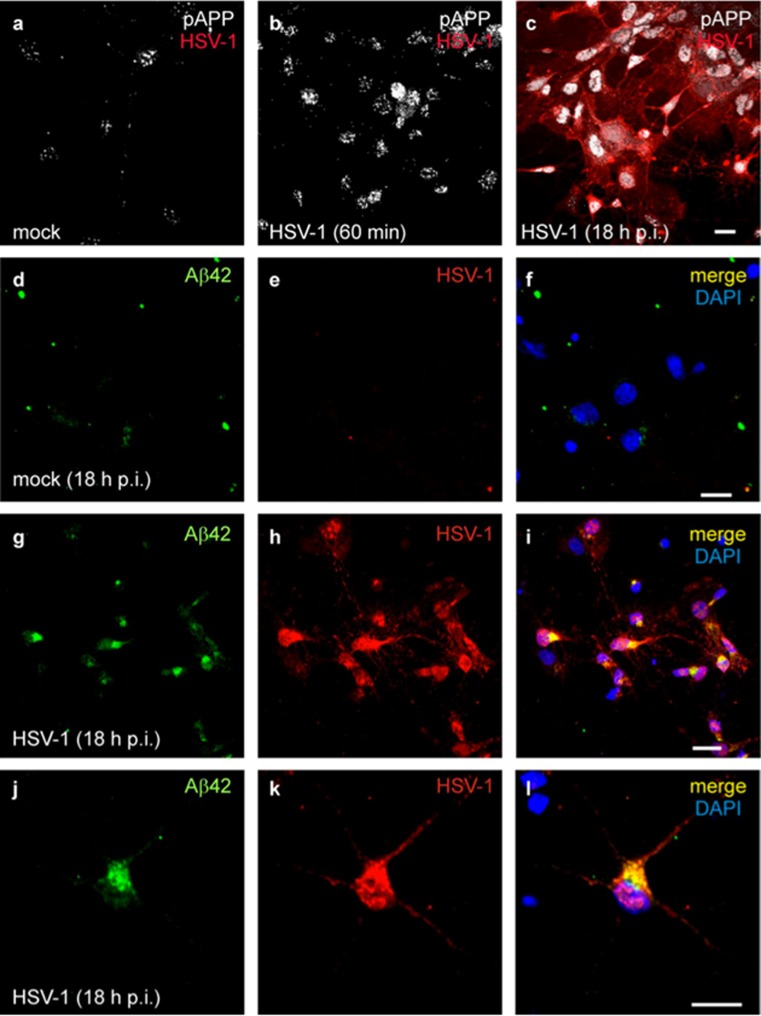
Effects of HSV-1 on APP phosphorylation and intracellular accumulation of amyloid-β protein (Aβ42) in cultured cortical neurons of E18 rats. **a–c** Time-dependent APP phosphorylation at threonine 668 induced by HSV-1; phosphorylated APP (pAPP) staining (*white*) in mock-infected cells (**a**), 1-h after cell challenge with HSV-1 (**b**) and 18 h post-infection (p.i.) (**c**). Neuronal infection is documented by HSV-1 labeling (*red*). **d–f**: no significant Aβ42 labeling (*green*) is observed in mock-infected cortical neurons. **g–i** Most HSV-1-infected neurons (*red*) exhibit Aβ42 immunoreactivity. **j–l**: high magnification image of a HSV-1-infected neuron showing marked Aβ42 staining. In **f**, **i** and **l**, the cell nuclei were stained with DAPI (*blue*). *Calibration bars*: 20 μm

The possible links between HSV-1 and Aβ are supported by experimental findings. Firstly, amyloid peptide is characterized by some degree of sequence homology with the HSV-1 glycoprotein B, and the viral protein has been suggested by some to act as a seed for Aβ deposits in amyloid plaques [100]. Moreover, new HSV-1 particles produced in the PNS have been proposed to recruit cell membranes containing APP, possibly during packaging in the Golgi apparatus [101], and to release APP during transport into the brain, thus contributing in some way to the formation of amyloid deposits. HSV-1 has also been shown to bind APP directly within the axonal transport into neurons [102, 103]. Secondly, Wozniak et al. [104] reported the accumulation of Aβ peptides in neurons and mouse brains infected with HSV-1, and then [58] the presence of the viral genome within amyloid plaques in AD brains. Other studies suggest that HSV-1 infection can interfere with APP processing: Shipley et al. [105] showed that HSV-1 infection of neuroblastoma cells induced the formation of a 55-kDa C-terminal fragment of APP; Wozniak et al. [104] found that BACE1 (β-secretase) and nicastrin (an essential component of the γ-secretase complex) immunolabeling is increased in the brains of HSV-1-infected mice. We recently reported that HSV-1 produces marked changes in neuronal excitability and intracellular Ca^2+^ signalling that cause APP phosphorylation and intracellular Aβ accumulation in rat cortical neurons [106]. We also demonstrated that HSV-1 triggers amyloidogenic cleavages of APP that are mediated in part by the action of β-secretase, γ-secretase and caspase-3-like enzymes, and that these result in the formation and intracellular accumulation of different APP fragments, including Aβ in both monomeric and oligomeric forms, with established potential for neurotoxicity [107] (Fig. 6). Finally, Cheng and colleagues recently demonstrated that intracellular HSV-1 interacts with APP, and that this interplay enhances viral transport and disrupts APP transport and distribution [108]. As for Aβ production, HSV-1 has been shown to cause the hyperphosphorylation of the microtubule-associated tau protein, thus impairing its intracellular transport functions. Zambrano et al. [109] first linked HSV-1 to tau hyperphosphorylation, showing evidence of altered microtubule dynamics and neurite damage occurring in HSV-1-infected cultures of murine cortical neurons. Subsequently, HSV-1 infection in neuroblastoma and glioblastoma cells was shown to induce the phosphorylation of tau at a number of sites that were shown to be phosphorylated in AD, also demonstrating a consistent increase in the amount of the relevant enzymes, i.e. glycogen synthase kinase 3β and protein kinase A [110]. More recently, Lerchundi and colleagues [111] showed evidence that HSV-1 induces the caspase-3-mediated cleavage of tau protein at its specific site (aspartic acid 421). This event has been associated with an increased kinetics of tau aggregation, observed in neurodegenerative pathologies. Overall, these findings support the idea that HSV-1 could contribute to neurodegeneration that characterizes age-associated pathologies such as AD.

#### HIV and Protein's Aggregates

There is a growing body of evidence indicating accelerated deposition of amyloid plaques also in HIV-infected brains. However, how HIV viral infection increases Aβ accumulation is poorly understood. It has been suggested that viral infection leads to increased production and impaired degradation of Aβ, mediated by upregulation of proinflammatory cytokine and inhibition of Aβ degrading enzyme, respectively [112–116]. Macrophages and microglia, both of which are productively infected by HIV in brains, play a pivotal role in Aβ degradation through the expression and execution of two endopeptidases, neprilysin (NEP) and insulin-degrading enzyme. It has been reported that HIV viral protein Tat-derived peptide inhibits NEP activity in vitro, and recombinant Tat added directly to brain cultures resulted in a 125 % increase in soluble Aβ oligomers [113, 116]. Recently, it was shown that monomeric Aβ degradation by primary cultured macrophages and microglia was significantly impaired by HIV infection due to an impairment in NEP endopeptidase activity, probably caused by the diminished transport of NEP to the cell surface and intracellular accumulation at the endoplasmic reticulum and in lysosomes [117]. The authors suggest that the malfunctioning of NEP in infected macrophages may contribute to the acceleration of β amyloidosis in HIV-infected brains, and propose modulation of macrophages as a potential preventive strategy for Aβ-related cognitive disorders in HIV-affected patients. Increased Aβ amyloidosis appears to be accelerated in brains of patients undergoing HAART [118]. The mechanisms through which HAART contributes to Aβ deposition are largely unknown. HAART is also reported to induce metabolic dysregulation, resulting in a hyperlipidemia, alterations in body fat distribution, diabetes, insuline resistance and coronary artery disease. Recently, Giunta and colleagues [119] provided evidence that antiretroviral treatment, especially when as combination, impairs microglial phagocytosis of β-amyloid and increases its production by neurons. HIV has also recently been shown to increase the level of hyperphosphorylated tau, through upregulation of its kinase CHK5, in a subset of HIV patients with the inflammatory condition known as HIV encephalitis [120]. A similar pattern of neurodegeneration, which includes increased brain levels of CDK5 and p35, alterations in tau phosphorylation and dendritic degeneration, was observed in transgenic mice engineered to express the HIV protein gp120. A previous paper by Giunta et al. [121] reported an increased amyloid-β deposition and tau hyperphosphodrylation in a mouse model of HIV-1 Tat-induced AD-like pathology generated by crossing PSAPP (APPswe and PSEN1dE9) [122] and HIV-1 Tat transgenic mice [123] compared with PSAPP mice, indicating that the viral protein Tat significantly promotes an AD-like pathology in PSAPP/Tat mice.

#### *C. pneumoniae* and Aβ Aggregates

Intranasal inoculation of *C. pneumoniae* (isolated from post-mortem brains of AD patients) has been shown to induce the formation of amyloid plaques in the brains of wild-type mice that increase in number as the disease progresses [67], together with intraneuronal Aβ42 accumulation. However, the bacterium effects on plaque formation have been contested by a subsequent similar work showing that a different strain of *Chlamydia* was unable to induce Aβ plaque formation in the brains of infected mice [124]. More recently, *Chlamydia* antigens have been detected alongside amyloid deposits and tau tangles in post-mortem AD brain tissue [68]. Further research is required to demonstrate definitely how both intracellular and extracellular amyloid aggregates and *C. pneumoniae* are interrelated.

Exposure of mammalian neuronal and glial cells and organotypic cultures to *Borrelia burgdorferi* spirochetes has been shown to produce amyloid deposits and tau hyperphosphorylation, indicating that bacteria and/or their degradation products may enhance the cascade of events leading to AD [125]. In this regard, it is noteworthy that spirochetes frequently co-infect with other bacteria and viruses, i.e. *C. pneumoniae* and HSV-1, suggesting that concurrent infections with several pathogens may also occur in AD. Finally, recent data reporting that Aβ peptides show an anti-microbial activity [126] and might act as a defence molecule of innate immunity which in some way support the hypothesis of the involvement of infectious agents in AD pathogenesis and aetiology: the accumulation of Aβ and plaque deposits may derive from an over-production of Aβ peptides directed against pathogenic neuroinvasions.

#### Influenza Virus and α-Synuclein Aggregates

The PD hallmark Lewy bodies are composed mainly of aggregated α-SYN. This protein aggregation has been shown to depend on the phosphorylation of a number of serine residues in α-SYN, particularly serine 129 [127]. Jang et al. [128] demonstrated that the highly infectious, neurotropic A/Vietnam/1203/04 (H5N1) influenza virus progresses from the peripheral nervous system into the CNS, where it can induce PD symptoms and the activation of microglia, a significant increase in phosphorylation and aggregation of α-SYN, which likely results in the observed substantia nigra pars compacta (SNpc) dopaminergic neuron degeneration 60 days after resolution of the infection.

### Alteration of Autophagic Processes

Autophagy is a highly conserved mechanism for the degradation and recycling of superfluous or damaged proteins as well as entire organelles that occurs mainly in cells under metabolic stress in order to provide an energy supply. This pathway is therefore essential for promoting health and longevity by regulating cellular survival, development, differentiation and homeostasis, as well as for defending cells from infectious intruders [129]. Different forms of autophagy (macroautophagy, microautophagy and chaperone-mediated autophagy) have been described as regulators of cell fate [130] and their differences depend on their physiological functions and the mode of cargo delivery to lysosomes. In particular, macroautophagy, the major regulated catabolic process of long-lived protein and organelle degradation, takes place within double-membrane vesicles, termed autophagosomes that deliver cytoplasmic cargo to lysosomes, whose hydrolases ensure the complete degradation of unwanted material. The formation of autophagosomes is directly catalyzed by LC3, a ubiquitin-like molecule, and is regulated by specific proteins of the yeast Atg family [131]. In particular, Beclin-1, the mammalian orthologue of yeast Atg6, has a central role in autophagy by interacting with several cofactors to regulate the biogenesis and maturation of autophagosomes as well as their fusion to lysosomes, thereby promoting autophagy.

The dysregulation of autophagy may contribute to a number of diseases, including aging, cancer, infectious diseases and neurodegenerative disorders [132–136]. In the latter case, defects in autophagy have been associated with the presence of aberrant proteins in the brain both in experimental models and in human patients. Mice engineered to suppress basal autophagy show neurodegenerative hallmarks, such as accumulation of inclusion bodies and intracellular protein aggregates in the brain [137, 138]. Increased numbers of autophagosomes have been found in AD, TSE, PD and HD brains where they may reflect the activation of autophagy as a physiological response to the disease or as a consequence of autophagosomal maturation defects, as in the case of AD [139–141]. Indeed, autophagy plays a key role in the clearance of aggregated proteins (oligomers and fibrils) associated with several neurodegenerative diseases, as they are poor substrates for proteasomal degradation. Acquired defects in autophagosome formation may result from the sequestration of autophagy proteins in the aggregates formed by mutant or misfolded proteins, as well as from defects in Beclin-1 and other autophagic proteins or from impaired delivery of autophagosomes to lysosomes due to mutation or alterations that affect the dynein motor machinery. Autophagy also targets RNA and DNA viruses and microbes, including herpes simplex virus, for sequestration and elimination [142–144], and this effect is particularly useful for long-lived cells such as neurons, in which it may represent a non-cytolytic mechanism of viral clearance. Moreover, autophagy may also exert a protective function during infection with neurotropic viruses, by promoting the survival of infected neurons and minimizing neuronal dysfunction. In addition, it is possible that autophagy may contribute to class II presentation of viral antigens by microglia or astrocytes during CNS infection. However, some infectious agents may overcome or take advantage of autophagy by replicating within autophagic vacuoles [145–148] or by inhibiting this process.

#### HSV-1 and Autophagic Pathways

HSV-1 encodes the ICP34.5 protein—infected cell polypeptide 34.5—that can disrupt the autophagic process [144, 149], thus protecting itself against destruction. This protein has also been shown to reverse the protein kinase R (PKR) defence mechanism, which is activated by the presence of virus-derived double-stranded RNA (dsRNA) and is aimed at shutting off cellular protein synthesis through the phosphorylation of elongation initiation factor 2α (eIF2α) [150]. Such a shutting-off would inhibit the synthesis of viral proteins, initiate apoptosis [151] and enhance autophagy. ICP34.5 mediates HSV1-induced eIF2α dephosphorylation [152], thus allowing viral protein synthesis. However, it uses two different mechanisms to antagonize host autophagy and PKR function. The N-terminal domain of ICP34.5, which is not required for the reversal of PKR-mediated eIF2α phosphorylation, binds to Beclin-1 and inhibits its autophagic function in autophagosome biogenesis [153], while the C-terminal domain of ICP34.5 (the GADD34 domain) recruits a host phosphatase, PP1α, to reverse PKR-mediated eIF2α phosphorylation and host cell translational shut-off [154]. These two mechanisms cooperate to block autophagy and may exacerbate the defective autophagy processes that characterize aging brains. Moreover, HSV-1-induced PKR activation in neuroblastoma cells and peripheral nervous tissue from infected mice has been suggested to increase the amyloidogenic APP processing and Aβ production by promoting BACE1 translation via eIF2α phosphorylation [155]. Interestingly, a recent paper from Santana et al. [156] shows that HSV-1 infection in neuroblastoma cells induces intracellular Aβ accumulation in autophagosomes, whose fusion with lysosomes appears to be aborted. As described above, HSV-1 has been proposed as a risk factor for AD, and APP, Aβ and the enzymes responsible for Aβ formation are present in autophagosomes in AD brains [157], which may constitute a reservoir of these pathogenic proteins. In this context, viral action via the ICP34.5 gene may prevent not only degradation of HSV-1 in the brain but also degradation of the aberrant cell proteins, including Aβ and hyperphosphorylated tau, thus possibly contributing to the formation of amyloid aggregates and neurofibrillary tangles.

#### HIV and Autophagosome Maturation

Like HSV-1, HIV has been shown to block autophagosome maturation, probably to prevent its own degradation. The autophagic step affected is autophagosome fusion to lysosomes. In particular, the viral protein Nef has been shown to inhibit autophagosome fusion with lysosomes by interacting with Beclin-1 and disabling its correct complex formation with the autophagic proteins that promote this fusion [158]. Moreover, HIV also appears able to inhibit neuronal autophagy. In particular, a recent study showed that products of microglia infected by the simian immunodeficiency virus (SIV, the HIV-analog that infects monkeys) inhibit neuronal autophagy, resulting in decreased neuronal survival, and that two major mediators of HIV-induced neurotoxicity, tumour necrosis factor-alpha and glutamate, had similar autophagy-reducing effects in neurons. Interestingly, the induction of autophagy in neurons through rapamycin treatment conferred significant protection to neurons in SIV-infected brains [159].

#### Influenza Virus and Authophagic Pathways

Influenza virus has also been reported to affect autophagy, causing autophagy activation in early phases of the infection in order to promote viral replication [160], while later it induces autophagosome accumulation in the cytoplasm of infected cells. Again, this latter event seems to be due to virus-induced defects in autophagosome maturation, achieved possibly via viral matrix protein 2, a proton-channel pumping unit, which binds ATG6/Beclin 1 [158, 161] and blocks the fusion between lysosomes and autophagosomes. The functional consequence of this block is a higher susceptibility of influenza A virus-infected cells to apoptosis. Our group [160] has recently demonstrated that pepstatin A, an inhibitor of cathepsin D that is involved in both autophagy and apoptosis [162], is able to decrease viral replication by interfering with this pathway. Interestingly, as previously reported [163], the influenza virus is able to induce apoptotic cell death in neuronal cells, even though they are characterized by a high expression of the antiapoptotic protein Bcl-2 and relatively low permissiveness to influenza virus replication, and it is possible that in this context a blocking of the autophagic process may enhance virus-induced apoptosis and thus neurodegeneration.

The reported findings suggest that autophagy is an important process that needs to be preserved in neurons exposed to insults that can lead to neurodegeneration, including viral infections.

### Oxidative Stress and Neurodegeneration

Several experimental findings point to oxidative stress as a key element in the pathogenesis of neurodegenerative diseases. The brain is particularly vulnerable to oxidative damage on account of its high oxygen levels, the presence of polyunsaturated fatty acids, transition metals in ionic form and low amounts of antioxidants [164]. In the elderly, where the redox state is unbalanced because of high levels of pro-oxidant species, the presence of metals such as iron ions in an oxygen-rich environment can induce further production of reactive oxygen species (ROS). The analysis at autopsy of brains from AD patients shows high levels of ROS and a drastic decrease in the intraneuronal content of glutathione (GSH), mainly in the hippocampus and cortex. These features also characterize the substantia nigra of PD patients, and are found in spinal fluid of patients with ALS [reviewed in 165]. This is associated with a high rate of cell membrane lipid peroxidation and nitration or oxidation of proteins and nucleic acids [166]. In particular, several of these oxidative events seem to be target-specific, such as for tyrosine nitration in α-SIN and in tau protein that are found in PD and AD, respectively [167, 168].

#### Viral Infections and Oxidative Stress

Viral infections are frequently associated with host-cell redox changes characteristic of oxidative stress [169–173].

##### HSV-1

HSV-1 infection induces an alteration of the intracellular redox state towards a pro-oxidant state through the depletion of GSH (the main endogenous antioxidant), the production of ROS, the induction of mitochondrial DNA damage, and endoplasmic reticulum (ER) stress [173–176]. Furthermore, HSV-1 infection in murine neuronal cells increases ROS levels and lipid peroxidation [177]. In agreement with these observations, high levels of lipid peroxidation products and protein nitrosylation were found in those brain areas where replicating or latent HSV-1 were detected after infection in primary sites [178, 179]. Overall, these data indicate the occurrence of oxidative damage in the brain following virus infection.

Alterations in the intracellular redox state towards a pro-oxidant state have been associated with formation of the typical histological alterations of brain tissue that occur in neurodegenerative diseases, including Aβ peptide generation and deposition [180–182]. HSV-1 was found to trigger multiple amyloidogenic APP processing mediated mainly by virus-induced upregulation of β- and γ-secretases. Interestingly, this event was prevented by antioxidant agents [107], suggesting that HSV-1-induced oxidative stress in neuronal cells may trigger β- and γ-secretase activation and, consequently, APP processing and Aβ formation. Some kinases involved in tau and APP phosphorylation [183–185] belong to the stress-activated protein kinase family, known to be activated during oxidative stress. A strong activation of these kinases has been observed both in post-mortem brains from AD patients [186, 187] and during in vitro HSV-1 infection [188, 189]. HSV-1 has been shown to induce AD-specific tau and APP phosphorylation, as well as the upregulation of the kinases involved in this event, such as GSK3β and PKA [106, 109, 110]. These effects may be further enhanced in vivo as consequence of oxidative stress response produced by microglial cells during HSV-1 infection [190].

##### HIV

Pioneer studies [191–193] showed evidence of oxidative stress markers in the cells and body fluids of HIV-infected patients. Garaci et al. [194] demonstrated that in vitro HIV infection significantly decreases the GSH content of human macrophages and suggested that this effect might be related to the preferential use of cysteine (the rate-limiting amino acid for GSH synthesis) for the synthesis of viral proteins. Interestingly, the brains of patients with HIV dementia show oxidative damage markers, including increased amounts of peroxynitrite, 4-hydroxynonenal and protein carbonyls [195]. The viral proteins gp120 and Tat have been shown to increase oxidative stress and to induce several damaging effects in neurons, including destruction of the cytoskeleton. These effects were reversed by antioxidants, suggesting an important role for oxidative damage in the pathogenesis of HIV dementia [196, 197]. Overproduction of superoxide anions and other free-radical species, possibly via the release of proinflammatory molecules by HIV-infected macrophages/microglial cells, has been suggested to play a role in the astroglial apoptotic cell death that characterizes HAD [198]. In this context, gp41, an envelope glycoprotein of HIV, has been shown to trigger inducible NO synthase (iNOS) expression and consequently NO production in human astrocytes and murine cortical brain cells in culture [199, 200], thus possibly contributing to the HAD pathogenesis.

##### Influenza Virus

Decreases in GSH levels and oxidative stress have also been found in cells and animals infected by influenza virus [172, 201–204]. Virus-induced pro-oxidant conditions play an important role in viral replication, by activating cellular kinases involved in nucleo-cytoplasmic traffick of viral proteins and by promoting the maturation of viral HA [163, 205].

Virus-induced oxidative stress has also been associated with the onset and progression of influenza virus-associated encephalopathy (IE) in murine models [reviewed in 206]. Markers of oxidative stress have been found in serum and CSF of patients with IE and have been proposed as possible predictive biomarkers of the severity of the disease [207]. As mentioned before, influenza virus infection has been related to the appearance of biochemical markers typical of PD [128], a neurodegenerative disease characterized by high levels of oxidative stress in substantia nigra and other brain regions. On the basis of these evidences, it is possible to speculate that virus-induced oxidative stress may play a role in this process. As a matter of fact, oxidative stress has shown to play a major role in the degeneration of the dopaminergic neurons that characterize PD [208]. The vulnerability of these neurons to oxidative stress was demonstrated by the ability of pro-oxidant molecules, such as 6-hydroxydopamine (6-OHDA), paraquat and rotenone to damage substantia nigra cells after stereotaxic or systemic administrations. Thus, chronic age-related oxidative stress [209] may result in the accumulation of misfolded proteins [210], through a direct oxidation of protein disulfide isomerse (PDI), an oxido-reductase involved in folding of glycoprotein. Specifically, the S-nitrosylation of PDI may play a central role in the progression of PD. Indeed, PDI S-nitrosylation disrupts its neuroprotective role of preventing neuronal cell death triggered by ER stress, the accumulation of misfolded proteins, or proteasome inhibition [211].

It is possible to speculate that an increased “oxidative burst” generated when the influenza virus infects aged neuronal cells may contribute to alterations in PDI function, increasing the production of misfolded proteins and contributing to the pathogenesis of PD.

### Synaptic Alterations

A large body of evidence suggests that deficits in synaptic transmission and plasticity play a major role in cognitive impairment and memory loss that characterize several neurodegenerative disorders including AD [212, 213]. Synaptic transmission occurs through chemical neurotransmitters that are released from pre-synaptic terminals in response to action potentials and bind specific receptors present on the post-synaptic side. The interaction between neurotransmitters and their post-synaptic receptors generates electrical signals (named postsynaptic potentials) that, when appropriately summed, may give rise again to action potentials thus allowing the propagation of information through the neural networks. Synaptic plasticity reflects the ability of a synaptic contact to change its efficiency (i.e. its signalling strength) depending on the activation pattern it previously experienced, and these plastic changes in information transmission are critical to learning and memory. However, the signal transmission at the synaptic level implies ion fluxes through the plasma membranes of both pre- and post-synaptic neurons that may have profound impact on neuronal functions and viability independently of electrical signal generation. A representative example is given by glutamate-dependent excitotoxicity, consisting of hyper-activation of calcium-permeable glutamate receptors (i.e. the *N*-methyl-d-aspartate receptors [NMDAR]) that produce intracellular calcium overload triggering pro-apototic pathways and leading to neuronal death [214, 215].

#### Virus and Synaptic Alterations

Viruses have been reported to significantly affect the synaptic function.

##### HIV

HIV-1 infection induces a progressive loss of synaptic connections in the CNS that appears in the early phases of the HAD pathology and correlates with the progression of the disease [216, 217]. In vivo studies demonstrated that some viral proteins are directly involved in these effects: mice engineered to express gp120 in astrocytes under an astrocyte-specific promoter show reduction of presynaptic terminals and dendrites in the CNS [218], whereas mice selectively expressing gp160 (the gp120 and gp141 heterodimers) in neurons exhibit synaptic dysfunctions [219]. Reportedly, gp120 induces synaptic damage through indirect and direct mechanisms involving NMDAR activation. Its indirect action is primarily mediated by soluble factors released from glial cells upon gp120 treatment [220–225]. In particular, gp120 activation of macrophages and microglia induces glutamate-related hyper-activation of NMDAR, with consequent ER stress and Ca^2+^ release [226]. Direct effects of gp120 on NMDA-evoked calcium influx involve modifications in the spatial location and density of NMDAR, possibly mediated by alterations of the biophysical properties of neuronal membranes induced by the viral protein that stabilizes the structure of lipid microdomains containing the receptor. This effect, together with gp-120-induced PKA- and PKC-dependent phosphorylation of NMDAR, results in a perturbation of the surface expression and spatial location of NMDARs [227].

NMDARs are also activated by the viral protein Tat [228, 229], resulting in Ca^2+^ rise [226, 230, 231] that in turn activates neuronal nitric oxide synthase (nNOS) leading to cell death [197]. Tat also affects neuronal dendritic structures causing proteasome-mediated degradation of microtubule-associated protein 2 (MAP2) and the collapse of cytoskeletal filaments [232]. Kim et al. [233] hypothesized that these effects, rather than the neuronal death, could account for the impaired synaptic plasticity observed in neural networks exposed to Tat [234]. Consistently, they found that the impaired network function and the decreased neuronal survival produced by Tat, both effects induced by LRP-dependent activation of NMDARs, result from distinct mechanisms that are mediated by the ubiquitin-proteasoma pathway and nNOS activation, respectively.

##### Influenza Virus

Influenza virus A nucleoprotein NP has also been shown to affect synaptic functions: rat hippocampal neurons exposed to recombinant NP, fused to 11–amino acid peptide transduction domain (PTD) of TAT, showed disturbances in postsynaptic functions, documented by reduced frequency and amplitude of the miniature excitatory postsynaptic currents. The authors hypothesized that the viral NP that localized in dendritic spinelike processes interferes with the expression or anchoring of postsynaptic glutamate receptors thereby disturbing synaptic functions [235].

##### HSV-1

Besides being specific target of viral proteins, synapses also represent a route through which viruses may travel between neurons, as this is the case of HSV-1 [236]. Consistently, nectin proteins, among which nectin-1 and nectin-2 that are used by HSV as virus entry and cell–cell spread mediator [237–240] have been involved in synapse formation [241]. We found that HSV-1 binding to neuronal membrane markedly affected the electrophysiological properties of rat cortical neurons and enhanced their excitability [106]. This effect consisted of persistent Na^+^ channel activation and K^+^ current inhibition leading to membrane depolarization and increased neuronal firing. Voltage-gated Ca^2+^ channel were consequently activated thus triggering intracellular Ca^2+^ signals raising the basal intracellular Ca^2+^ levels. Calcium signals potently promoted APP phosphorylation and processing with consequent intracellular and extracellular accumulation of several neurotoxic fragments including Aβ oligomers [106, 107]. These virus-induced APP fragments might induce synaptic dysfunction resembling that underlying the cognitive deficits observed in AD.

### Apoptosis

Neuronal cell death by apoptosis, a highly organised process characterized by chromatin condensation, shrinkage of the nucleus and cytoplasm, DNA fragmentation and disintegration of the cell into small apoptotic bodies that are destined to be phagocytized [242], underlies the symptoms of many neurodegenerative diseases. Apoptotic death of hippocampal and cortical neurons results in AD symptoms such as memory loss and cognitive decline; the death of dopaminergic neurons and the consequent apoptosis of midbrain neurons that use dopamine as a neurotransmitter lead to the characteristic tremors of PD; the loss of striatal neurons that control body movements characterizes HD; apoptosis of motor neurons in the spinal cord is responsible for the manifestations of ALS.

Several neurotropic viruses induce apoptosis in neural cells and these effects can contribute to the pathogenesis of the virus-induced disease. Other viruses prevent apoptotic death of host cells in order to estabilish a persistent infection. On the other hand, programmed cell death may also be used by the host as a non-inflammatory response aimed at removing the virus. Thus, the fate of infected cells depends on a complex network of interactions between a virus and its host cell, which has yet to be fully delucitated.

#### HSV-1

During strong and acute infection, such as occurs in HSE, HSV-1 induces neuronal cell death, although apoptosis does not occur during later sequelae even when inflammation is still present [243], and is likely the cause of long-term entorhinal cortex and hippocampal cell loss along with memory deficits in mice [244]. Additionally, encephalitic HSV-1 has been reported to induce apoptosis in hippocampal neurons through the activation of JNK pathways [245]. On the other hand, the presence of HSV-1 DNA in many healthy brains [11] suggests that the virus does not necessarily provoke cell death in cerebral tissue. In this regard, viral latency-associated transcript (LAT) has been reported to inhibit apoptosis [246], particularly by blocking the two major mammalian apoptotic pathways, i.e. the extrinsic apoptotic pathway and the intrinsic pathway [247–249], to promote neurite sprouting in neuroblastoma cells and to protect C1300 and N2A cells from killing by CD8 T lymphocytes in vitro [250, 251].

Based on these findings and others showing that LAT play a key role in the HSV-1 latency-reactivation cycle [252–254], the authors proposed that by preserving latently infected neurons from apoptotic cell death, it supports HSV-1 reactivation rates and spread into neuronal tissue. Viral protein expressed during productive infection with HSV-1 can also induce or inhibit apoptosis in a cell type-dependent manner following the infection of cultured cells [255–259]. In particular, experiments performed with genetically modified viruses showed that the apoptotic pathway induced by HSV-1 in neuronal cells within the first hour of infection could be blocked at multiple steps of the viral replicative cycle. The viral proteins synthesized between 3 and 6 h post-infection [260] and also some viral glycoproteins such as glycoproteins D and J, which are involved in the virus entry, have a role in this suppression of apoptosis in HSV-1-infected neuronal cells [261]. Moreover, the R1 subunit of viral ribonucleotide reductase inhibits apoptosis in hippocampal neurons and in differentiated PC12 cells through upregulation of the anti-apoptotic protein Bag-1 expression and activation of the ERK and Ras/MEK/MAPK pathways [262, 263].

In agreement with these findings, we also were unable to detect apoptotic markers in HSV-1-infected rat cortical neurons up to 24 h p.i.. However, when we challenged primary rat cortical neurons with the supernatants of neuroblastoma cells infected with HSV-1 in the presence or absence of β- and γ-secretase inhibitors, we found that infected-culture supernatants triggered apoptosis in these cells, even when the cultures were exposed to UV light, which inactivates the virus they contain. The infected supernatants obtained in the presence of β- and γ-secretase inhibitors exhibited much lower neurotoxicity. These findings suggest that supernatants of HSV-1-infected cells are highly neurotoxic for primary neurons, and that this effect is related to the presence of virus-induced APP fragments released into the extracellular medium rather than to the presence of “active” viral particles [107]. Nevertheless, they indicate an apoptotic pathway indirectly activated by the virus and mediated mainly by virus-induced Aβ peptides. Carter [264] addresses the hypothesis that activation of the immune system by other pathogens, i.e. those implicated in AD (e.g. *Helicobacter pylori, C. pneumoniae* and others [265]) might disturb the fragile balance between HSV-1-and host neuronal cells, allowing viral destruction, but causing neuronal loss. Activation of the immune system thus appears to be a potent inducer of neuronal death via inflammatory mediators [266].

#### *C. pneumoniae*

*C. pneumoniae* has been reported to inhibit the apoptotic process following infection in different celly types, including neutrophils [267], monocytes and epithelial cells [268–271], microglial cells [124] and neuronal cells [272]. When the latter were infected with *C. pneumoniae*, they appeared resistant to staurosporine-induced apoptosis. In particular, *C. pneumoniae* infection downregulated pro-apoptotic cytoplasmic proteins such as cytochrome c released from mithocondria and activated caspase-3/7, which are normally upregulated following staurosporine treatment. However, considering that they detected *C. pneumoniae* in AD brains [8, 273, 274], the authors hypothesized that, as for other cell types [275], this bacterium could both inhibit apoptosis and promote neuronal death by necrosis. This could also account for the inflammatory process activated by the pathogen. The ability of *C. pneumoniae* to inhibit the apoptotic process could result in chronic or prolonged infection in the CNS that, by promoting amyloidogenesis and neuroinflammation, may contribute to the neuropathogenesis of AD.

#### HIV

HIV infection reportedly induces apoptosis and neuronal loss in the CNS [276–279], but the mechanisms underlying these events have yet to be clarified. Multiple viral proteins, including gp120 and tat, have been involved in virus-induced neuronal loss. Cell death similar to that described in the brains of HAD patients has been detected in cerebral regions of transgenic mice expressing gp120, suggesting that the HIV glycoprotein is able to induce apoptosis alone [218]. A large body of evidence supports this notion, showing that gp120 causes degeneration and death of several types of neurons maintained in culture. However, it is still unknown whether gp120 is able to induce neuronal apoptosis directly or indirectly. Some findings support the hypothesis that gp120 infects infiltrating macrophages and lymphocytes, causing the release of pro-inflammatory and neurotoxic cytokines, such as IL-6, thus damaging sensory neurons and producing neuropathies [280]. Other data support the hypothesis that gp120 may interact directly with neurons, which indeed express both the gp120 co-receptors CXCR4 and CCR5 [281, 282]. In particular, gp-120-induced apoptosis has been demonstrated to be mediated mainly following its interaction with CXCR4 [283, 284], as demonstrated both in vivo and in vitro studies. For example, treatment of cultured human or rat neurons with gp120 leads to neuronal apoptosis [285–288] and intracerebroventricular injection of HIV-1 gp120 in rats produces apoptotic neuronal death in vivo [289–291].

Tat, a transactivating nuclear regulatory protein that is critical for viral replication, is released by infected macrophages and microglia and can be taken up by neighbouring cells, including neurons, where it can express its pro-apoptotic potential [292]. This action is inhibited by neurotrophic factors, including brain-derived neurotrophic factor (BDNF) and nerve growth factor [292], which activate the transcription factor NF-kB and upregulate the expression of the anti-apoptotic protein Bcl-2. Interestingly, long-term incubation of Tat in cultured PC12 cells causes decreased expression and activity of the transcription factor CREB, which in turn plays a key role in neuronal survival through the upregulation of BDNF [293]. Thus neurotoxic HIV proteins are able to activate pro-apoptotic cascades and to inhibit pro-survival pathways. However, together with other neurotoxic viral proteins such as Vpr, gp140 and Nef [294–296], other endogenous factors released from infected cells, especially macrophages, contribute to HIV-induced neuronal apoptosis, including excitatory amino acids, NO, MMPs and proinflammatory cytokines such as TNF-a and SDF-1 [297–299]. Moreover, in HIV-1-infected patients that are also drug abusers, the activation of opioid receptors, widely expressed by astrocytes and astrocyte precursors, can induce apoptosis, thus combining with the viral effects to accelerate the progression of HAD.

#### Influenza Virus

The influenza virus is also known to induce apoptosis in infected cells through other mechanisms. Among these, we previously demonstrated that in neurons, influenza virus activates the mitochondrial (intrinsic) apoptotic pathway through p38MAPK-mediated phosphorylation of Bcl-2 [163]. The interaction between Bcl-2 and this kinase diminishes the ability of Bcl-2 to prevent the cell undergoing virally induced apoptosis, but it also reduces the ability of the virus to replicate effectively. The immediate result is programmed death of infected cells and the release of a relatively low number of infective virions. These data let us hypothesize that despite the low virus replication of neuronal cells [172] the apoptotic signals activated by the virus, may contribute to the onset of neurodegeneration.

It has previously been reported that the avian H5N1 type of influenza A virus can be detected in neurons and astrocytes of human brains at autopsy [300, 301]. Recently, Ng et al. [302] demonstrated that the H5N1 virus can infect human astrocytic and neuronal cells, resulting in the induction of direct cellular damage and pro-inflammatory cytokine cascades. Indeed, increased expression of IL-6 and/or TNF-α mRNA was detected in both astrocytic and neuronal cells (human glioblastoma and neuroblastoma cells) at 6 and 24 h p.i.. TNF-a treatment induced apoptosis, as well as pro-inflammatory cytokine, chemokine and inflammatory responses in differentiated cells.

The recent immunohistochemical study in PD performed by Rhon and Catlin [303] revealed that the presence of influenza virus within the SNpc in post-mortem PD brain sections was associated with apoptotic oligodendrocytes labelled by the Beclin-1 caspase-cleavage product antibody (BeclinCCP) in the white matter of the SN of PD and DLB (dementia with Lewy body) patients. Many of the oligodendrocytes labelled with the BeclinCCP antibody displayed hallmark features of apoptosis, including fragmentation of processes and shrunken cell bodies. This degeneration of oligodendrocytes, which are known to play a key role in the myelination of axons in the CNS, may contribute to the extrapyramidal symptoms associated with PD.

Apoptosis has also been identified in the brains of influenza encephalopathy and encephalitis patients [304]. Microglial cells were markedly increased in TUNEL-positive influenza encephalopathy and encephalitis brains compared with TUNEL-negative brains. Immunoreactivity for active-caspase 3, demonstrated by immunohistochemistry, and the overexpression of a caspase-cleaved fragment of poly(ADP-ribose) polymerase, indicated that activation of caspase 3 is involved in the apoptotic pathway in the brains of influenza encephalopathy patients.

## Conclusions

**Fig. 7:**
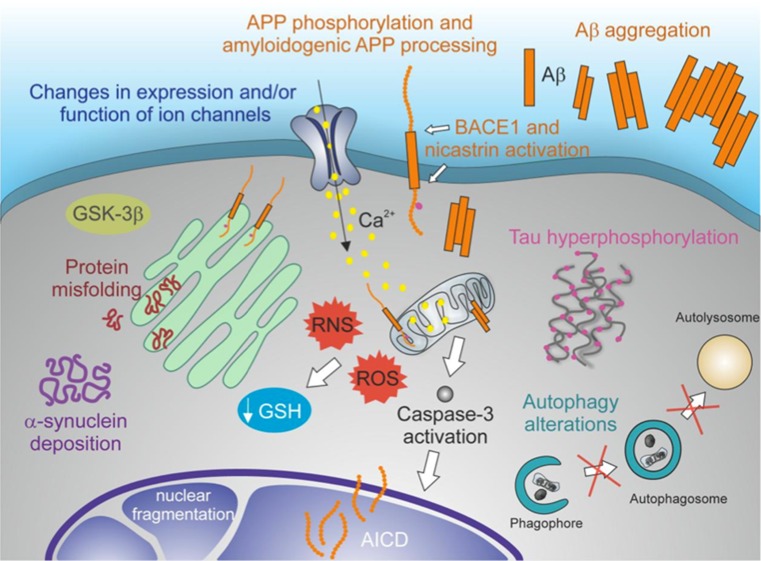
Molecular hallmarks of neurodegeneration induced by infectious agents in neurons of the central nervous system (for further details, see text)

Advances in microbiological research have led to in-depth understanding of the structure and replication mechanisms of several pathogens, as well as of their interactions with host cells. However, a growing body of evidence suggests that when an infectious agent reaches sites different from those of its primary replication it may produce mild infections that eventually cause additional and unexpected effects. This is the case for persistent CNS infections caused by continuous pathogen replications (e.g. HIV and *C. pneumoniae* infections), repeated infections (e.g. influenza virus) or latent infections followed by life-long reactivations (as in the case of HSV-1). Repeated cycles of pathogen replication within the CNS produce functional and molecular hallmarks of neurodegeneration, including protein misfolding, deposition of misfolded protein aggregates, alterations of autophagic pathways, oxidative stress, neuronal functional alterations and apoptotic cell death (Fig. 7). These effects accumulate over time, thus contributing to neurodegeneration. The pathogen-induced effects add to and are possibly amplified by several factors such as metabolic disorders, genetic alterations and other environmental risk factors, involved in the pathogenesis of neurodegenerative diseases. As a result, the pathogen-induced damage amplifies and accelerates the neurodegenerative process, whose signs are usually manifested during aging.

The data reviewed in our paper suggest that more detailed understanding of the molecular mechanisms underlying pathogen-mediated neuronal damage may pave the way to the identification of new preventive and/or therapeutic strategies aimed at counteracting the progression of these devastating pathologies.
